# Three-Year Clinical Impact of Murray Law-Based Quantitative Flow Ratio and OCT- or FFR-Guidance in Angiographically Intermediate Coronary Lesions

**DOI:** 10.1161/CIRCINTERVENTIONS.123.013191

**Published:** 2024-04-25

**Authors:** Cristina Aurigemma, Daixin Ding, Shengxian Tu, Chunming Li, Wei Yu, Yingguang Li, Antonio Maria Leone, Enrico Romagnoli, Rocco Vergallo, Alessandro Maino, Carlo Trani, William Wijns, Francesco Burzotta

**Affiliations:** Fondazione Policlinico Universitario A. Gemelli IRCCS, Rome, Italy (C.A., E.R., R.V., C.T., F.B.).; Lambe Institute for Translational Research, Smart Sensors Laboratory and Curam, University of Galway, Ireland (D.D., W.W.).; Department of Cardiology, Ren Ji Hospital, School of Medicine, Shanghai Jiao Tong University, China (D.D., S.T.).; Biomedical Instrument Institute, School of Biomedical Engineering, Shanghai Jiao Tong University, China (S.T., C.L., W.Y., Y.L.).; Ospedale Fatebenefratelli Isola Tiberina Gemelli Isola Roma, Italia (A.M.L.).; Università Cattolica del Sacro Cuore, Rome, Italy (A.M., C.T., F.B.).

**Keywords:** fractional flow reserve, optical coherence tomography, percutaneous coronary intervention, quantitative flow ratio

## Abstract

**BACKGROUND:**

The FORZA trial (FFR or OCT Guidance to Revascularize Intermediate Coronary Stenosis Using Angioplasty) prospectively compared the use of fractional flow reserve (FFR) or optical coherence tomography (OCT) for treatment decisions and percutaneous coronary intervention (PCI) optimization in patients with angiographically intermediate coronary lesions. Murray law-based quantitative-flow-ratio (μQFR) is a novel noninvasive method for the computation of FFR. In the present study, we evaluated the clinical impact of μQFR, FFR, or OCT guidance in FORZA trial lesions at 3-year follow-up.

**METHODS:**

μQFR was assessed at baseline and, in the case of a decision to intervene, after (FFR- or OCT-guided) PCI. The baseline μQFR was considered the final μQFR for deferred lesions, and post-PCI μQFR value was taken as final for stented lesions. The primary end point was target vessel failure ([TVF]; cardiac death, target-vessel-related myocardial infarction, and target-vessel-revascularization) at a 3-year follow-up.

**RESULTS:**

A total of 419 vessels (199 OCT-guided and 220 FFR-guided) were included in the FORZA trial. μQFR was evaluated in 256 deferred lesions and 159 treated lesions (98 OCT-guided PCI and 61 FFR-guided PCI). In treated lesions, post-PCI μQFR was higher in OCT-group compared with FFR-group (median, 0.93 versus 0.91; *P*=0.023), and the post-PCI μQFR improvement was greater in FFR-group (0.14 versus 0.08; *P*<0.0001). At 3-year follow-up, OCT- and FFR-guided treatment decisions resulted in comparable TVF rate (6.7% versus 7.9%; *P*=0.617). Final μQFR was the only predictor of TVF. μQFR ≤0.89 was associated with 3× increase in TVF (11.6% versus 3.7%; *P*=0.004). PCI was a predictor of higher final μQFR (odds ratio, 0.22 [95% CI, 0.14–0.34]; *P*<0.001).

**CONCLUSIONS:**

In vessels with angiographically intermediate coronary lesions, OCT-guided PCI resulted in comparable clinical outcomes as FFR-guided PCI. μQFR estimated at the end of diagnostic or interventional procedure predicted 3-year TVF.

**REGISTRATION:**

URL: https://www.clinicaltrials.gov; Unique identifier: NCT01824030.

**WHAT IS KNOWN**

- The management of patients with angiographically intermediate coronary lesions is a daily clinical challenge. Once the decision to perform percutaneous coronary intervention is made, percutaneous coronary intervention may be optimized using adjunctive devices such as intracoronary imaging or functional techniques.
- Murray law-based quantitative-flow-ratio is a novel method for fast computation of fractional flow reserve from a single angiographic projection, thus providing noninvasive information about both hemodynamic significance of coronary lesions and optimization of percutaneous coronary intervention result in the case of treatment.

**WHAT THE STUDY ADDS**

- In patients with angiographically intermediate coronary lesions, an optical coherence tomography–based management (treatment decision and optical coherence tomography-optimization in the case of percutaneous coronary intervention performance) has a 3-year clinical impact that looks comparable with the gold-standard fractional flow reserve.
- Novel methodology for fast computation of fractional flow reserve from coronary angiography may help predict the 3-year outcome of angiographically intermediate coronary lesion managed by fractional flow reserve or optical coherence tomography.

The management of angiographically intermediate coronary lesions (AICLs) is a daily clinical challenge for interventional cardiologists. Percutaneous coronary intervention (PCI) driven by functional assessment of coronary stenoses is associated with a better clinical outcome as compared with angiography.^1–4^ Indeed in current European Society of Cardiology (ESC) and European Association for Cardio-Thoracic Surgery (EACTS) guidelines on coronary revascularization and chronic coronary syndromes, invasive functional assessment is recommended to evaluate stenosis before revascularization, unless very high-grade stenosis.^5,6^ The use of intracoronary imaging techniques, such as optical coherence tomography (OCT), has a promising impact on PCI optimization,^7–10^ whereas its role in the decision-making of lesion treatment is still debated. The FORZA trial (FFR or OCT Guidance to Revascularize Intermediate Coronary Stenosis Using Angioplasty; NCT01824030) is the first single-center prospective, randomized trial comparing the clinical and economic implications of FFR or OCT in the management of patients with AICLs.^11^ In the trial, imaging guidance by OCT was associated with an increased rate of coronary revascularization at 1-month follow-up with a significant increase in administered contrast, contrast-induced acute kidney injury, and total costs.^12^ However, at 13-month follow-up, OCT guidance was associated with a lower incidence of the composite of major adverse cardiac events or significant angina and target vessel failure (TVF).^13^

Murray law-based quantitative-flow-ratio (μQFR) is a novel method for fast computation of FFR from a single angiographic projection,^14^ thus providing noninvasive information about both hemodynamic significance of coronary lesions and optimization of PCI results in the case of treatment. In the present study, we assessed μQFR in FORZA trial vessels undergoing OCT or FFR-guided procedures and evaluated its impact on 3-year TVF.

## METHODS

The authors are open to providing access to the data upon reasonable request.

### Population

Data for this analysis was obtained from the FORZA trial dataset. The FORZA trial enrolled 350 patients with stable ischemic heart disease or stabilized (culprit lesion treated previously) acute coronary syndrome and evidence of at least 1 AICL, a coronary lesion in the nondistal segment of major epicardial vessel with a visually estimated percentage diameter stenosis between 30% and 80%. Patients with AICLs were randomized 1:1 to either FFR guidance or OCT guidance for both PCI performance and, in the case of revascularization, PCI optimization.^11^ When the enrolled patient had multiple AICLs, all lesions were managed using the same technique (OCT or FFR according to randomization). The study protocol was approved by the local Ethical Committee (internal code: 6261/13), and recruited patients gave their consent to participate in this study. Coronary revascularization was performed when FFR was ≤0.80 or when at least one of the following OCT criteria was present: (1) area stenosis ≥75%; (2) area stenosis between 50% and 75% and minimal lumen area <2.5 mm^2^; and (3) area stenosis between 50% and 75% and plaque rupture. In the FFR-guided PCI arm, the aim was the achievement of a poststenting FFR≥0.90. An optimal OCT result was defined as absence of stent malapposition (defined as distance between strut and vessel wall >350 or <350 and >200 µm for a length >600 µm), major underexpansion (in-stent minimal cross-sectional area <75% of the reference lumen area), or major edge dissection (defined as length >600 µm).

### Computation of Baseline- and Post-PCI μQFR

For the purpose of this analysis, all coronary angiography of FORZA trial procedures was retrospectively reviewed, and μQFR of AICLs was assessed at baseline and, in the case of coronary revascularization, after (FFR- or OCT-guided) PCI. The μQFR analyses were performed using the AngioPlus Core software (Version V3, Pulse Medical, Shanghai, China) by an experienced analyst, who had obtained the official certification for μQFR analysis and was blinded to patients’ clinical and outcome data. Details of the acquisition of angiography, OCT, and FFR evaluation have been reported previously.^11–13^ For μQFR analysis, the angiographic projection with the best exposure of the interrogated lesion, and with minimal overlap and foreshortening of the interrogated lesion, was selected.^14,15^ In the case of bifurcation lesions, in addition to the above criteria, we preferably selected the projection with full exposure of the ostial of side branches. This allows for accurate delineation of the contours of both the main vessel and side branches, contributing to more accurate reconstruction of step-down reference vessels for μQFR computation. The cutoffs for abnormal μQFR were ≤0.80 at baseline evaluation and ≤0.89 after PCI.^16^

For each vessel, the postprocedural (after either diagnostic coronary angiography in the case of treatment deferral or after PCI in the case of FFR- or OCT-guided intervention) value of μQFR (defined as post-PCI μQFR in the case of treated vessels and baseline μQFR for deferred vessels) was highlighted as final μQFR since it reflects the estimated hemodynamic relevance of the coronary disease left in the vessel after the initial invasive management.

### Study End Points

The primary outcome was a 3-year TVF defined as the composite of cardiac death, target vessel–related myocardial infarction, and subsequent target vessel revascularization (TVR), which was analyzed on a per-vessel basis. Cardiac death was defined as death due to cardiac causes, including cardiac arrest, myocardial infarction, low-output failure, or fatal arrhythmia. Target vessel–related myocardial infarction was defined as spontaneous myocardial infarction related to the index lesion. Subsequent TVR was defined as any PCI or coronary artery bypass surgery of an index lesion. All outcomes of interest were confirmed using source documentation collected at each hospital and were centrally adjudicated by an independent clinical events committee.

### Statistical Analysis

Continuous variables were tested for normal distribution by Shapiro-Wilk test and are reported as mean±SD if normally distributed or as median (interquartiles) if non-normally distributed. Comparison of continuous parameters was performed by Student *t* test if normally distributed, and by Mann-Whitney *U* test if non-normally distributed. Categorical variables were reported as counts (percentage) and were compared using χ^2^ or Fisher exact test.

Data were analyzed on a vessel level. The time-to-first event rates for each subgroup were estimated using Kaplan-Meier methods followed by a log-rank test. Between-group differences were estimated by hazard ratio (HRs) with 95% CIs using a Cox proportional hazards model. Tests for proportional hazards of each covariate were based on scaled Schoenfeld residuals. Sensitivity analyses of the prognostic value of final μQFR in predicting 3-year TVF were performed by multivariable Cox regression in 2 models: model 1 included patient ID as a random effect to account for the clustering effect within the same patients, model 2 included baseline covariates (interrogated lesion in the left anterior descending artery, age, female sex, diabetes, and acute coronary syndrome) as fixed effects and patient ID as a random effect. As baseline functional disease severity is likely to affect the PCI-associated achievable physiological gain, in the subgroup of lesions treated with PCI, baseline µQFR was adjusted in the Cox proportional hazards model. Landmark analysis was performed at 13 months, the time point which had been used for the primary end point of the FORZA study, for evaluation of the association of final μQFR in predicting 3-year TVF and separate end points. The area under the receiver-operating characteristic curve was used to test the predictive value of final μQFR in differentiating adverse events. The optimal cutoff value of final μQFR in predicting 3-year TVF was derived from the receiver-operating characteristic curve by maximizing the sum of sensitivity and specificity. Patient and vessel characteristic variables were studied in terms of their predictive value in determining final μQFR ≤0.89 using logistic regression.

Statistical significance was defined as *P*<0.05. All statistical analyses were performed with SPSS version 25 (SPSS Inc, Chicago, IL) and Stata version 15.0 (StataCorp, College Station, TX).

## RESULTS

### Baseline Clinical and Lesion Characteristics

**Figure 1.**
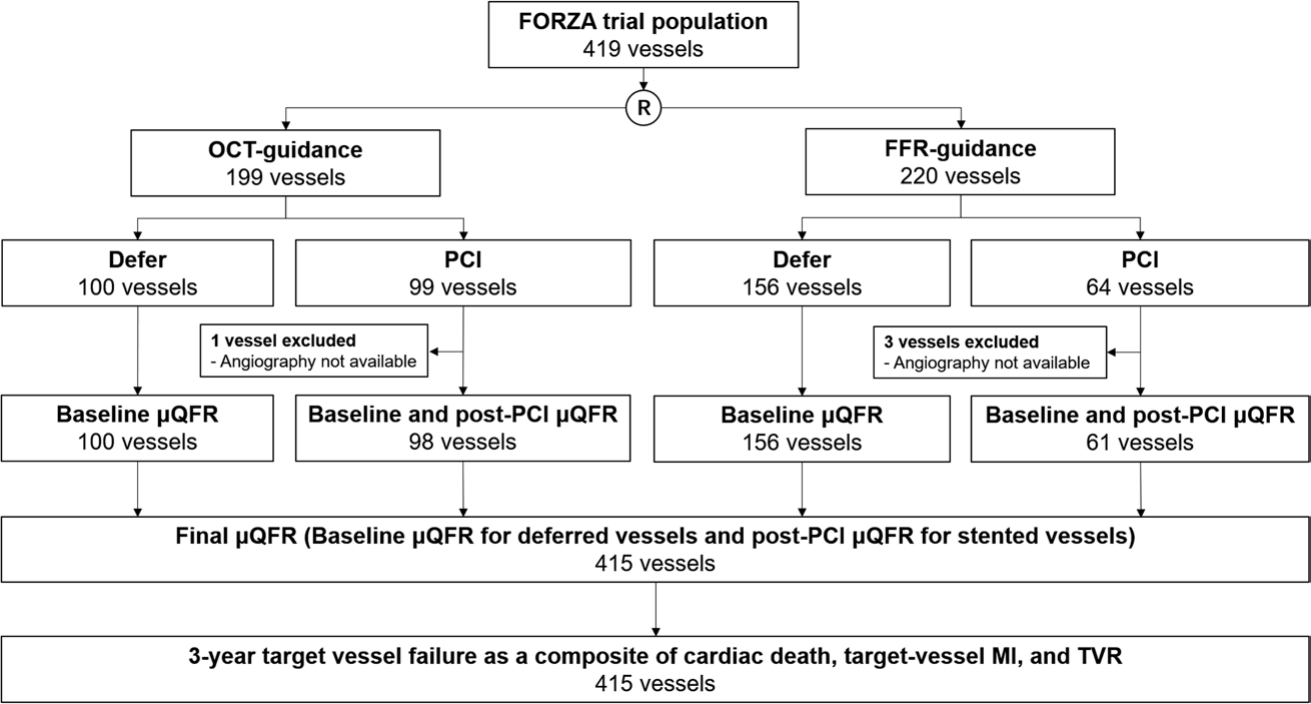
**Study flow chart.** μQFR indicates Murray law–based quantitative flow ratio; FFR, fractional flow reserve; FORZA, Fractional Flow Reserve vs Optical Coherence Tomography to Guide Revascularization of Intermediate Coronary Stenoses; MI, myocardial infarction; OCT, optical coherence tomography; PCI, percutaneous coronary intervention; and TVR, target vessel revascularization.

In the FORZA trial population, 199 vessels were randomized to the OCT imaging arm and 220 vessels to the FFR arm. More vessels were managed with PCI in OCT (49.7%, 99 out of 199) compared with in FFR arm (29.1%, 64 out of 220; *P*<0.0001; Figure 1). In this study, we have evaluated AICLs. Therefore, the distribution of FFR value is not normal.

After excluding 4 vessels due to missing angiography, baseline μQFR and post-PCI μQFR were successfully analyzed in 98 treated vessels in the OCT group, and in 61 treated vessels in the FFR group. Baseline μQFR was successfully analyzed in all 256 deferred vessels. In total, the final μQFR was available for 415 vessels from 347 patients.

Baseline patient characteristics are reported in Table 1, and baseline vessel characteristics are reported in Table 2. Of note, the majority (65.8%) of interrogated vessels were left anterior descending artery, and median baseline μQFR was 0.86 as expected for real AICLs.

**Table 1.**
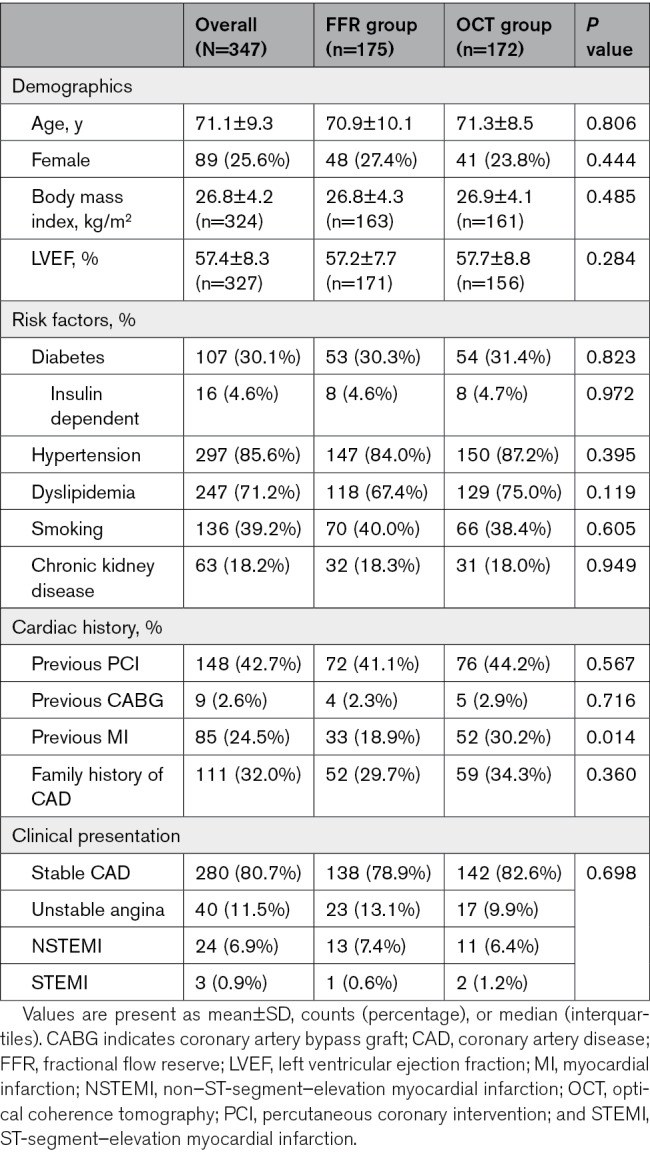
Baseline Patient Characteristics

**Table 2.**
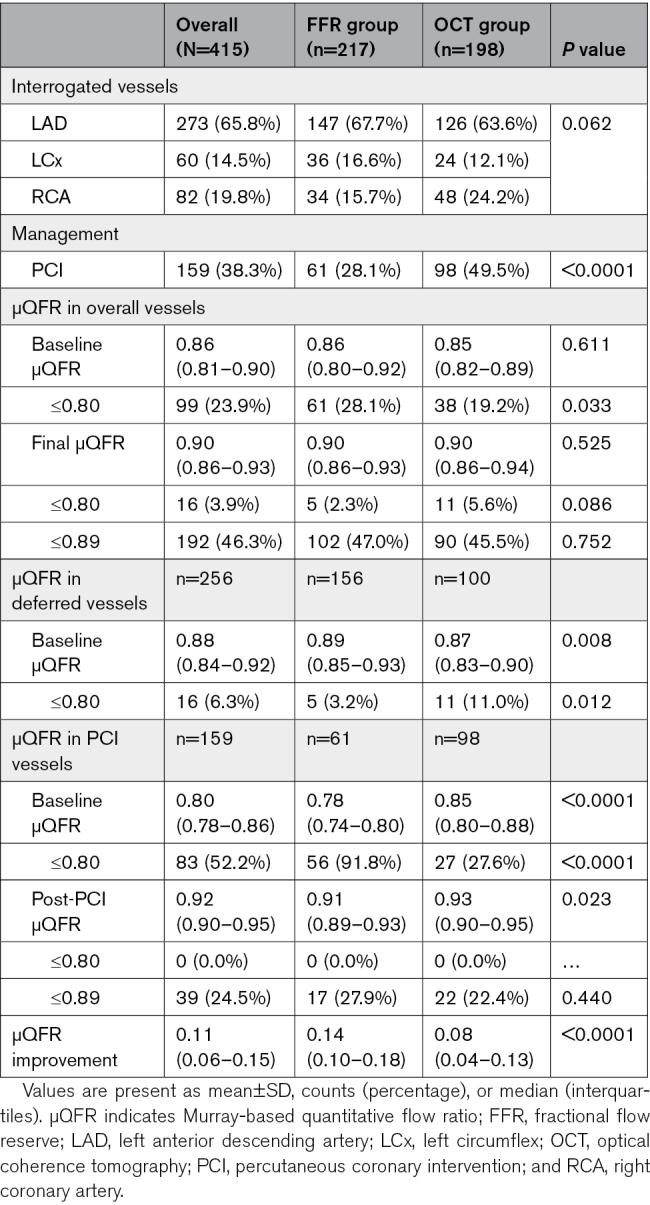
Baseline Vessel Characteristics

### Distribution of μQFR

Baseline μQFR was 0.86 (0.81–0.90) in overall population and was similar between the 2 randomization arms: 0.86 (0.80–0.92) in FFR group and 0.85 (0.82–0.89) in OCT group, *P*=0.611. The correlation of pre-PCI FFR and μQFR and the diagnostic performance of pre-PCI μQFR ≤0.80 in predicting pre-PCI FFR ≤0.80 are reported in Figure S1 and Table S1. Compared with deferred vessels, treated vessels had significantly lower baseline μQFR (0.80 [0.78–0.86] versus 0.88 [0.84–0.92]; *P*<0.0001), which improved significantly after PCI (0.92 [0.90–0.95]; *P*<0.0001). Post-PCI μQFR was significantly lower in FFR-guided procedures as compared with OCT-guided (0.91 [0.89–0.93] versus 0.93 [0.90–0.95]; *P*=0.023), whereas μQFR improvement from baseline to final post-PCI was significantly higher in FFR-guided procedures (0.14 [0.10–0.18] versus 0.08 [0.04–0.13]; *P*<0.0001).

Overall, the final μQFR was 0.90 ([0.86–0.93]; Figure S2), and the rate of final μQFR≤0.89 according to randomization was 47% (median, 0.90 [0.86–0.93]) in FFR-guided group and 45.5% (median, 0.90 [0.86–0.94]) in OCT-guided group. Among 39 treated vessels with final µQFR ≤0.89, 37/39 (95%) vessels had pressure gradients predominantly out-of-stent. The rates of final μQFR ≤0.80 and ≤0.89 obtained in FFR and OCT groups are reported in Table 2.

### Predictors of Final μQFR

As detailed in Table S2, among all variables listed in Tables 1 and 2, PCI and interrogated lesion in left anterior descending artery were only predictors for final μQFR≤0.89. PCI was a significant predictor of higher final μQFR (odds ratio, 0.22 [95% CI, 0.14–0.34]; *P*<0.001), while interrogated lesion in left anterior descending artery was a significant predictor of lower final μQFR (odds ratio, 2.40 [95% CI, 1.57–3.67]; *P*<0.001).

### 3-Year Clinical Outcomes: Impact of Guidance Modality and μQFR

All patients completed 3-year follow-up. Within 3 years, cardiac deaths were observed in 4 patients. Target vessel myocardial infarction and TVR occurred in 7 and 24 vessels, respectively. In total, TVF occurred in 7.2% of all vessels and, numerically, fewer TVF occurred in treated versus deferred vessels (5.0% versus 8.6%; *P*=0.190; Table 3).

**Table 3.**
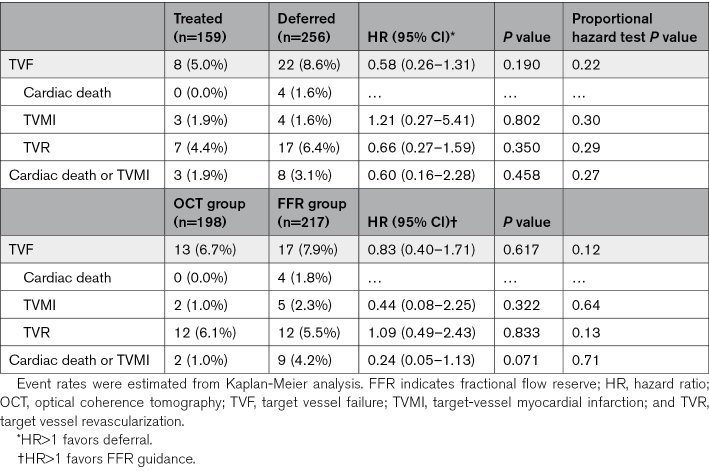
Three-Year Clinical Outcomes in Treated and Deferred Vessels, in OCT, and FFR Groups

**Figure 2.**
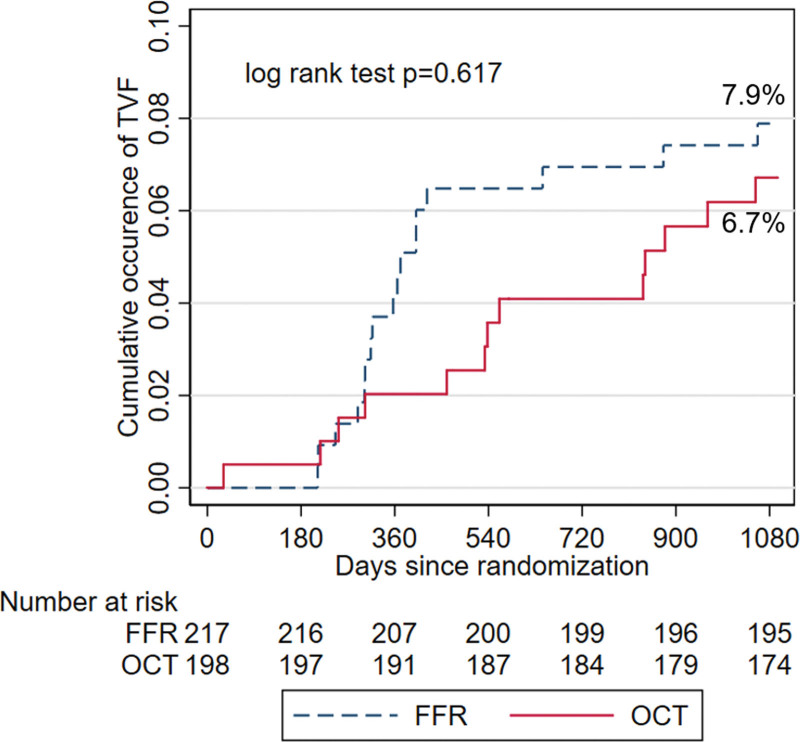
**Cumulative occurrence of 3-y target vessel failure (TVF) in optical coherence tomography (OCT) and fractional flow reserve (FFR) groups.** Three-year TVF occurrence was comparable in OCT vs FFR group (6.7% vs 7.9%; hazard ratio, 0.83 [95% CI, 0.40–1.71]; *P*=0.617).

Over 3 years, TVF occurred in 6.7% of the OCT group and 7.9% of the FFR group (HR, 0.83 [95% CI, 0.40–1.71]; *P*=0.617; Figure 2). Of note, a trend towards fewer cardiac deaths or target-vessel myocardial infarction was observed in the OCT group (1.0% versus 4.2% in the FFR group; *P*=0.071; Table 3).

**Figure 3.**
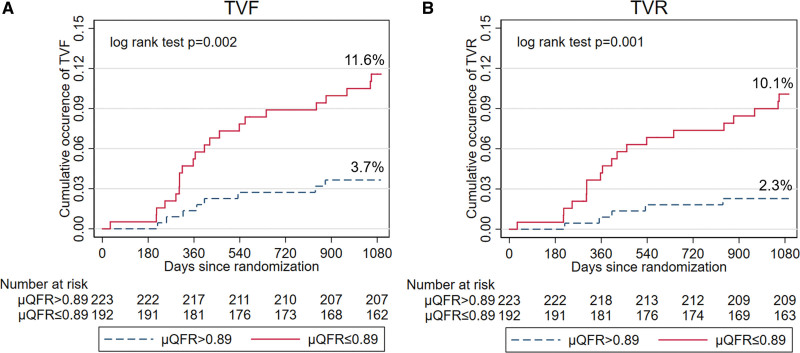
**Cumulative occurrence of 3-y target vessel failure (TVF) and target vessel revascularization (TVR) in high and low final Murray law-based quantitative flow ratio (μQFR) groups.** Final μQFR values ≤0.89 was associated with significantly higher 3-yTVF (**A**) rate compared with those with values >0.89 (11.6% vs 3.7%; hazard ratio [HR], 3.29 [95% CI, 1.47–7.39]; *P*=0.004), driven by significantly higher TVR (**B**) rate (10.1% vs 2.3%; HR, 4.57 [95% CI, 1.71–12.25]; *P*=0.002).

**Figure 4.**
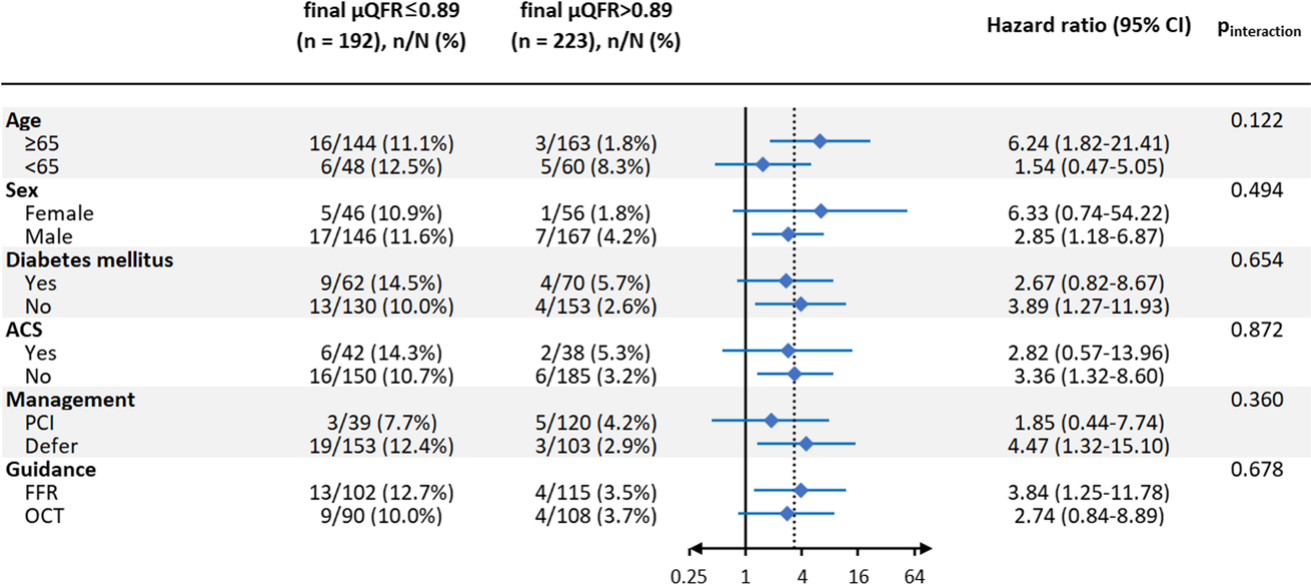
**Subgroup analyses for final Murray law–based quantitative flow ratio (μQFR) in predicting target vessel failure (TVF).** Prognostic value of final μQFR values ≤0.89 in predicting 3-y TVF rate was consistent across subgroups. ACS indicates acute coronary syndrome; FFR, fractional flow reserve; OCT, optical coherence tomography; and PCI, percutaneous coronary intervention.

Among the variables listed in Tables 1 and 2, final μQFR was the only independent predictor of 3-year TVF (HR for per 0.10 increase: 0.55 [95% CI, 0.30–0.98]; *P*=0.044; Table S3). Vessels with final μQFR values ≤0.89 had significantly higher 3-year TVF rate compared with those with values >0.89 (11.6% versus 3.7%; HR, 3.29 [95% CI, 1.47–7.39]; *P*=0.004; Figure 3; Table 4). The predictive value of final μQFR≤0.89 remained after multivariable adjustment (Table S4) and was consistent across subgroups, with no significant interaction between FFR and OCT, or between treated and deferred vessels (Figure 4; Tables S5 and S6). The higher TVF rate in vessels with final μQFR≤0.89 was driven by higher rate of TVR (10.1% versus 2.3%; HR, 4.57 [95% CI, 1.71–12.25]; *P*=0.002; Figure 3; Table 4). Thirteen-month landmark analysis showed that final μQFR≤0.89 was predictive of TVF both at 13 months (HR, 3.24 [95% CI, 1.03–10.19]; *P*=0.044) and afterward until 3-year follow-up (HR, 3.34 [95% CI, 1.06–10.49]; *P*=0.039), with no significant interaction between the 2 time periods (*P* value for interaction, 0.60; Figure S3). As for separate end points, final μQFR≤0.89 was predictive of TVR at 13 months and afterward until 3 years (Table S7).

**Table 4.**
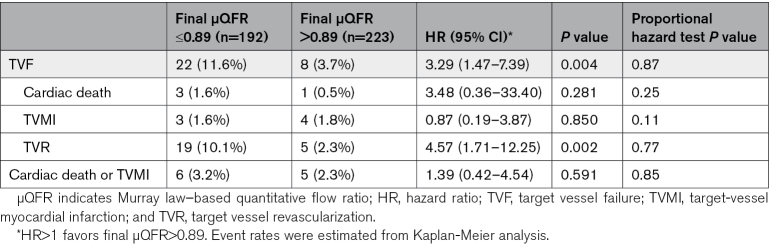
Three-Year Clinical Outcomes Stratified by Final μQFR

Receiver-operating characteristic curve analysis identified a final μQFR cutoff of ≤0.89 as having the best predictive accuracy for 3-year TVF (area under receiver-operating characteristic curve, 0.64 [95% CI, 0.60–0.69]; *P*=0.004) and for TVR (area under receiver-operating characteristic curve, 0.68 [95% CI, 0.63–0.73]; *P*<0.001; Figure S4).

## DISCUSSION

**Figure 5.**
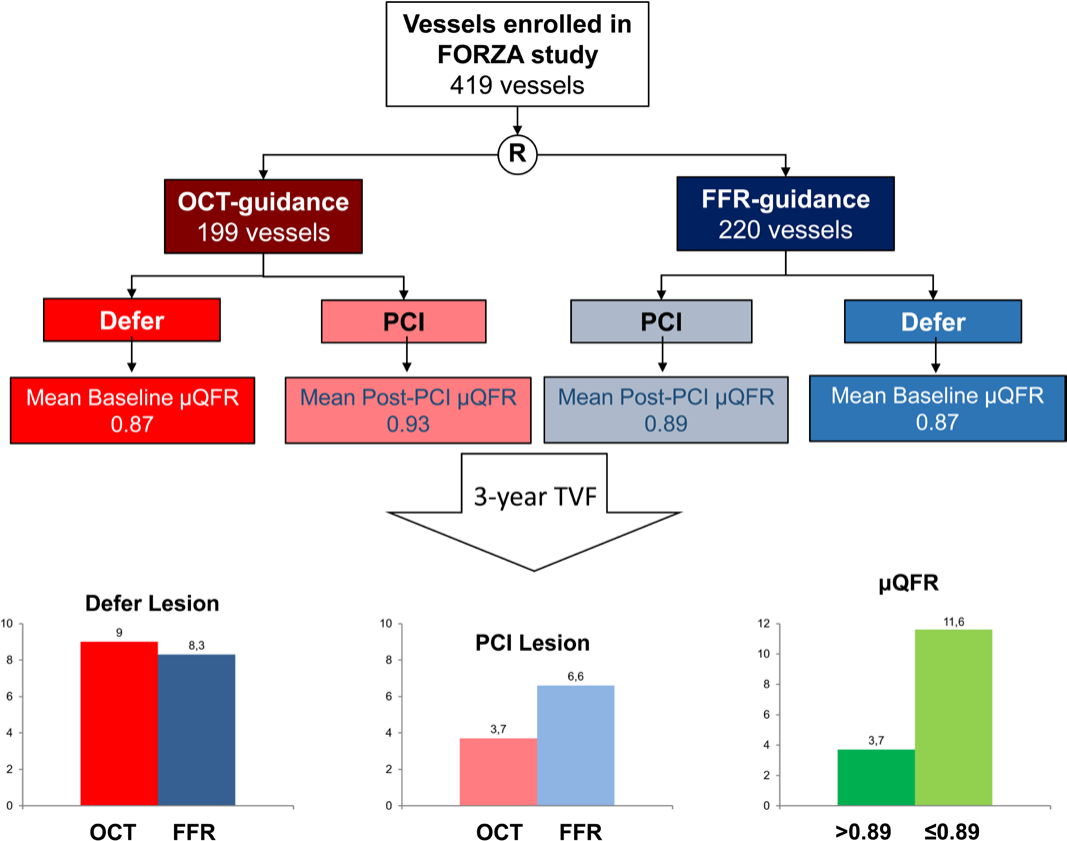
**Central Illustration.** The main original findings of the clinical impact of Murray law–based quantitative flow ratio (μQFR), fractional flow reserve (FFR), or optical coherence tomography (OCT) guidance in FORZA trial (Fractional Flow Reserve vs Optical Coherence Tomography to Guide Revascularization of Intermediate Coronary Stenoses) lesions at 3-y follow-up are (1) OCT and FFR-guided treatment decision resulted in comparable 3-y target vessel failure (TVF) rate; (2) the postprocedural (after either percutaneous coronary intervention [PCI] or coronary angiography in the case of treatment deferral) μQFR was the only clinical predictor of TVF at 3 y; (3) OCT-guided PCI is associated with higher post-PCI μQFR as compared with FFR-guided, while FFR-guided PCI is associated with a higher uQFR improvement compared with OCT-guided PCI.

The management of patients with ischemic heart disease with AICLs represents a daily clinical challenge and once the decision to perform PCI is made, PCI optimization using adjunctive devices such as intracoronary imaging or functional techniques is debated. For instance, novel processing tools for angiographic images are becoming available and might offer novel opportunities to guide both revascularization decisions and PCI optimization. In the present study, we applied a novel noninvasive method for fast computation of FFR from a single angiographic projection (μQFR) and reported for the first time the 3-year clinical outcomes observed in the FORZA trial. The main original findings are provided in Figure 5:

1. OCT- and FFR-guided treatment decisions resulted in comparable 3-year TVF rate
2. the postprocedural (after either PCI or coronary angiography in the case of treatment deferral) μQFR was the only clinical predictor of TVF at 3 years
3. OCT-guided PCI is associated with higher post-PCI μQFR as compared with FFR-guided, while FFR-guided PCI is associated with a higher uQFR improvement compared with OCT-guided PCI

The role of coronary physiological assessments utilizing pressure-derived FFR in decision-making treatment is ascertained.^5^ PCI optimization using FFR is less certain but several studies have demonstrated that post-PCI FFR values are inversely associated with adverse cardiac events.^17–21^ In the TARGET-FFR (Trial of Angiography Versus Pressure-Ratio-Guided Enhancement Techniques—FFR), a physiology-guided incremental optimization strategy was associated with further intervention in 30.5%. Although an FFR-guided optimization strategy did not significantly increase the proportion of patients with a final post-PCI FFR ≥0.90, an FFR-guided optimization strategy was associated with a lower proportion of cases with post-PCI FFR ≤0.80.^20^ In the FORZA study, despite the study protocol recommendation, an FFR evaluation was not obtained in all FFR-guided PCI and the rate of further intervention was only 14%.^12^ This issue could have concurred to determine the lower post-PCI μQFR observed in FFR as compared with the OCT arm. The rate of further stent optimization was low in the FORZA study population and is partially reflected by 24.5% of vessels with suboptimal post-PCI µQFR ≤0.89. Among vessels with final µQFR ≤0.89, the pressure gradient was predominantly out-of-stent. This finding underlines that even if PCI optimization had been done for these cases, the final physiology is less likely to be improved and in this case an appropriate pre-PCI planning based on a specific endotype of pressure gradients might be more useful for achieving better post-PCI results. The use of intracoronary imaging techniques, such as OCT, has an increasingly recognized favorable impact on PCI optimization.^7–10,22,23^ In the FORZA trial, OCT guidance, as compared with FFR, triggered a significant increase of PCI on AICLs^12^ which were conducted with OCT-based further interventions in 34%. Such OCT-associated increased invasiveness (higher number of PCIs, higher rates of interventions to optimize PCI result) resulted in better μQFR (both post-PCI or final) and did not jeopardize the clinical outcomes at 3-year follow-up. Indeed, at 3-year follow-up, the incidence of TVF was 5% in treated lesions and 8.6% in deferred lesions. Of note, studies reporting on the long-term outcome of OCT-based PCI are lacking and the TVF rate observed in the FORZA trial compares favorably with the 6.6% reported in the 3-year follow-up of imaging-guided arm of the ULTIMATE trial.^24^ Recently, ILLUMIEN IV data have demonstrated that OCT-guided PCI does not offer a benefit in terms of TVF compared with angio-guided PCI in the setting of complex coronary artery lesions.^25^ Indeed, in the ILLUMIEN IV trial, the rate of TVF is higher (7.5%) than the TVF (5%) reported in our study, due to a different risk degree of investigated coronary lesions. In our study, we enrolled angiographically intermediate stenosis, which the vast majority treated with medical therapy alone. Furthermore, up to 3 years, the overall rate of TVF continued to be numerically (albeit not significantly) lower in OCT versus FFR, thus suggesting that OCT guidance has the potential to offer a valuable alternative to physiological guidance. Extended follow-up duration and dedicated larger studies are deserved to better establish the patients and lesions that might benefit more from an initial physiological or invasive imaging approach.

One of the main novelties in the field of coronary angiography interpretation is represented by the potential to process the acquired images and, using computational models, derive noninvasive evaluations of coronary physiology.^26,27^ Such an approach has started to be evaluated in clinical trials, and we tested its potential in the context of the data collected in a prospective randomized trial focused on AICL. We confirmed the feasibility of μQFR evaluation in such a context and found a promising clinical signal since the only clinical predictor of 3-year TVF was post-μQFR for stented lesion (post-PCI OCT-derived parameters were not evaluated) and pre-μQFR for deferred lesions. In particular, a μQFR≤0.89 was associated with 3× increase in TVF.

### Study Limitations

The methodology used in the present study to generate μQFR was retrospectively applied and the high overall feasibility noticed might have been facilitated by the fact that all coronary angiographies, collected within the framework of a study, were conducted in the setting of a prospective trial. During the trial, after randomization, the decision to test individual vessels/lesions was left to operator’s discretion so that it is impossible to rule out the possibility that some device-related bias could have caused the slight excess of vessels investigated by FFR. Furthermore, the enrolled population is a low-risk population, including patients with angiographic intermediate stenosis, which the vast majority treated with medical therapy alone. Therefore, our results will need to be confirmed in more complex patient population. In the present study, we were not able to assess the association of final µQFR with residual angina because residual symptoms by the Seattle Questionnaire have not been systematically assessed at a 3-year follow-up.

### Conclusions

The present FORZA trial substudy reporting the 3-year clinical outcomes of enrolled patients and adding original μQFR assessments supports the safety of an initial OCT-guided approach in AICL and provides promising insights regarding the potential novel methods for fast computation of FFR from coronary angiography.

## ARTICLE INFORMATION

### Sources of Funding

This work was supported by the Natural Science Foundation of China (grant no. 82020108015 and 81871460) and by Shanghai Jiao Tong University (grant no. YG2023ZD24) to Dr Tu, by Science Foundation Ireland Research Professorship Award to Dr Wijns (grant no. 15/RP/2765).

### Disclosures

Dr Wijns reports institutional research grants and past honoraria from MicroPort; senior advisor of Rede Optimus Research, Corrib Core Laboratory and co-founder of Argonauts, an innovation facilitator. Dr Tu is a co-founder of Pulse Medical and reports research grant and consultancy from Pulse Medical. Drs Burzotta, Trani, and Aurigemma received speaker’s fees from Abbott, Medtronic, Abiomed and Terumo. Dr Leone received speaking honoraria from St. Jude Medical/Abbott, Medtronic, Abiomed and from Bracco Imaging. The other authors report nop conflicts.

### Supplemental Material

Figures S1–S4

Tables S1–S7

## Supplementary Material


