# Supplementary material for: Three-Year Clinical Impact of Murray Law-Based Quantitative Flow Ratio and OCT- or FFR-Guidance in Angiographically Intermediate Coronary Lesions
Source: Circ Cardiovasc Interv. 2024 Apr 25;17(5):e013191. doi: 10.1161/CIRCINTERVENTIONS.123.013191 (PMC11268551; doi:10.1161/CIRCINTERVENTIONS.123.013191)
Supplement: Supplementary file 1 [file hcv-17-e013191-s001.pdf]

Supplemental Materials

Supplemental Figure S1. Correlation and agreement of pre-PCI FFR and μQFR......2
Supplemental Figure S2. Distribution of final μQFR at a vessel level 3
Supplemental Figure S3. Landmark analysis at 13-months for final μQFR ≤0.89 in predicting 3-year TVF at a vessel-level 4
Supplemental Figure S4. Receiver-operating characteristic curves for final μQFR in predicting 3-year TVF (A) and TVR (B) at a vessel level 5
Supplemental Table S1. Diagnostic performance of pre-PCI μQFR ≤0.80 in predicting pre-PCI FFR ≤0.80 (220 vessels)..6
Supplemental Table S2. Predictors of final μQFR ≤0.89 7
Supplemental Table S3. Predictors of 3-year TVF 8
Supplemental Table S4. Sensitivity analysis of final μQFR in predicting 3-year TVF and TVR...9
Supplemental Table S5. 3-year clinical outcomes in high and low final μQFR groups stratified by randomization arm 10
Supplemental Table S6. 3-year clinical outcomes in high and low final μQFR groups stratified by vessel treatment 11
Supplemental Table S7. Landmark analysis at 13-months for final μQFR ≤0.89 in predicting 3-year TVF and separate endpoints at a vessel-level.................................12

Supplemental Figure S1. Correlation and agreement of pre-PCI FFR and μQFR

Pre-PCI FFR and pre-PCI μQFR had good agreement (mean difference 0.00 ± 0.06, p = 0.88) and moderate correlation (r = 0.66, p<0.0001).

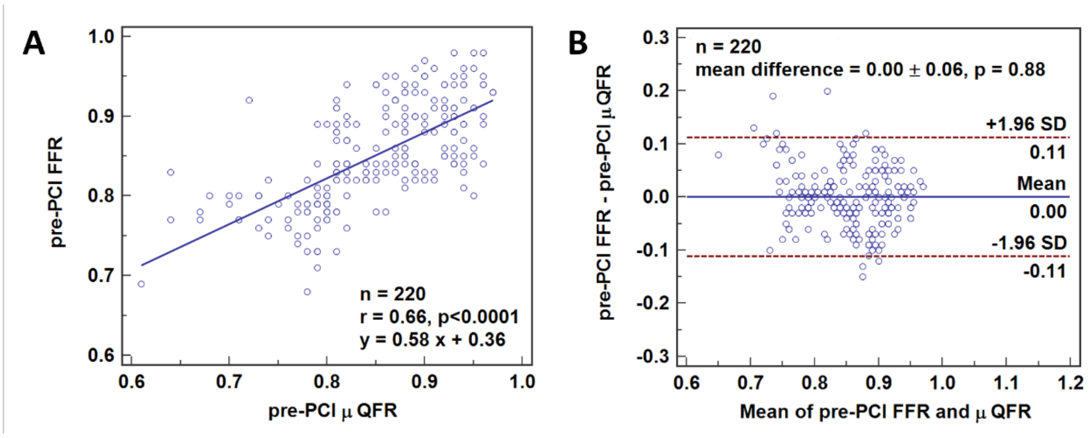

Supplemental Figure S2. Distribution of final µQFR at a vessel level

Final µQFR (post-PCI µQFR for stented vessels and baseline µQFR for deferred vessels) had a median value of 0.90 [0.86 – 0.93].

µQFR, Murray law-based quantitative flow ratio.

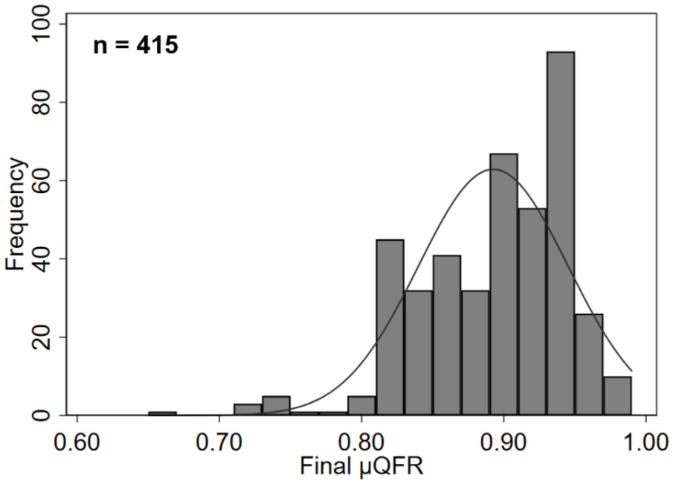

Supplemental Figure S3. Landmark analysis at 13-months for final µQFR ≤0.89 in predicting 3-year TVF at a vessel-level

Final µQFR ≤0.89 was predictive of TVF both during the first 13 months (HR 3.24 [95%CI 1.03-10.19], p = 0.044) and afterwards until 3-year follow-up (HR 3.34 [95%CI 1.06-10.49], p = 0.039), with no significant interaction between the two time periods (p for interaction 0.60).

HR, hazard ratio; TVF, target vessel failure; µQFR, Murray law-based quantitative flow ratio.

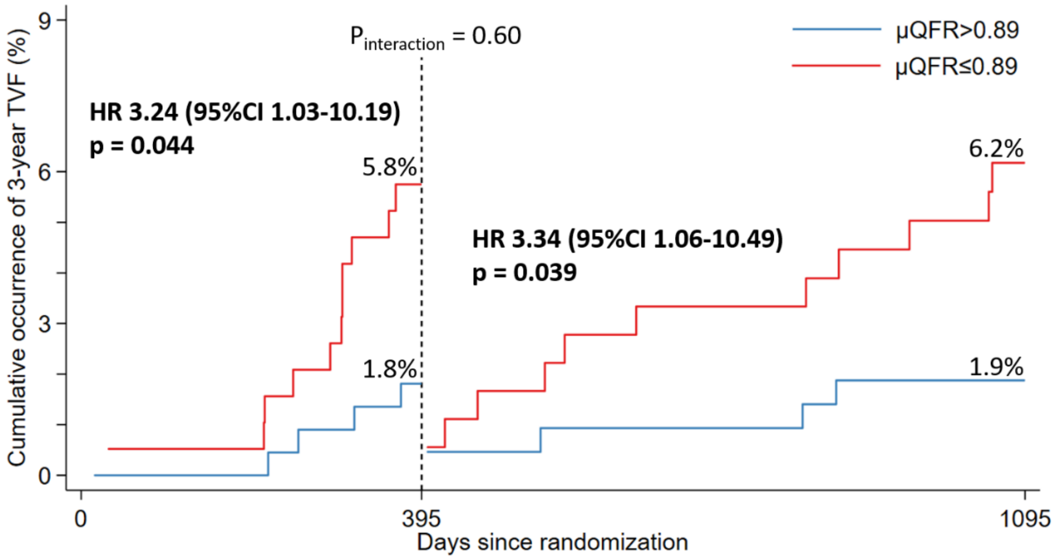

Supplemental Figure S4. Receiver-operating characteristic curves for final μQFR in predicting 3-year TVF (A) and TVR (B) at a vessel level

AUC, area under receiver-operating characteristics curve; TVF, target vessel failure; TVR, target vessel revascularization; μQFR, Murray law-based quantitative flow ratio.

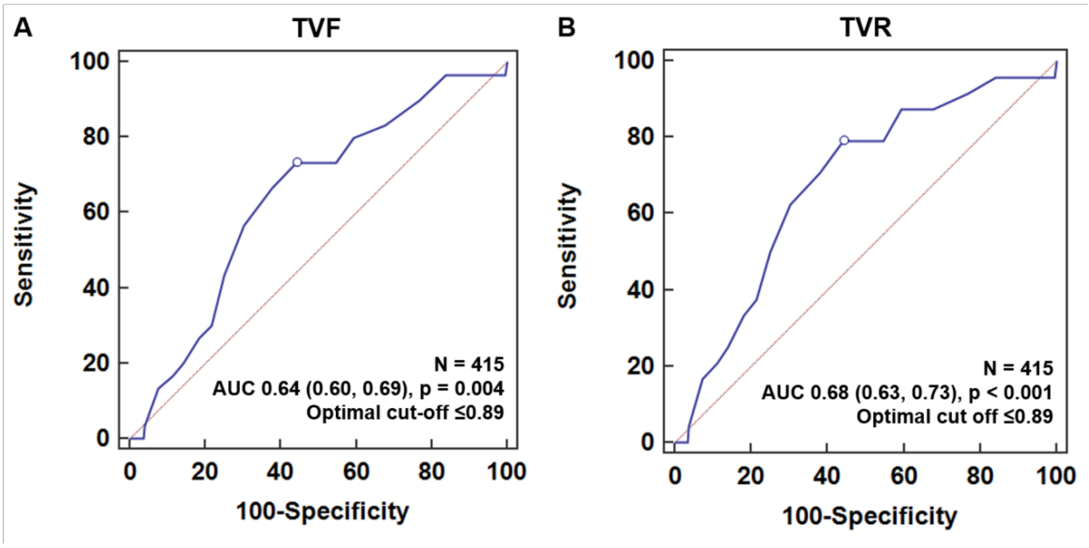

Supplemental Table S1. Diagnostic performance of pre-PCI μQFR ≤0.80 in predicting pre-PCI FFR ≤0.80 (220 vessels)

Using a pre-PCI FFR ≤0.80 for identifying physiologically significant stenosis, the accuracy of pre-PCI μQFR ≤0.80 was 92.3% (95%CI: 88.7% – 95.8%), with a sensitivity of 86.7% (95%CI: 75.4% – 94.1%) and a specificity of 94.4% (95%CI: 89.6% – 97.4%).

	**pre-PCI μQFR ≤0.80**
Accuracy, % (95%CI)	92.3 (88.7, 95.8)
Sensitivity, % (95%CI)	86.7 (75.4, 94.1)
Specificity, % (95%CI)	94.4 (89.6, 97.4)
PPV, % (95%CI)	85.2 (73.8, 93.0)
NPV, % (95%CI)	95.0 (90.3, 97.8)
+LR, (95%CI)	15.4 (8.1, 29.3)
-LR, (95%CI)	0.14 (0.07, 0.3)

Supplemental Table S2. Predictors of final µQFR ≤0.89

Variables	OR (95% CI)	P
Age, years	1.006 (0.99, 1.02)	0.528
Female	0.94 (0.60, 1.47)	0.785
Body mass index, kg/m^2	1.01 (0.96, 1.06)	0.689
Multivessel disease	0.76 (0.50, 1.15)	0.193
LVEF, %	1.004 (0.98, 1.03)	0.721
Diabetes mellitus	1.04 (0.69, 1.58)	0.844
Insulin dependent Diabetes mellitus	1.17 (0.45, 3.01)	0.745
Hypertension	0.98 (0.57, 1.68)	0.931
Dyslipidemia	0.81 (0.53, 1.23)	0.325
Smoking	0.85 (0.57,1.26)	0.421
Chronic kidney disease	1.49 (0.92, 2.43)	0.106
Previous PCI	0.86 (0.58, 1.26)	0.550
Previous CABG	0.43 (0.11, 1.63)	0.213
Previous MI	0.81 (0.52, 1.26)	0.350
Family history of CAD	0.95 (0.62, 1.44)	0.795
Stable CAD	0.73 (0.45, 1.20)	0.214
Interrogated lesion in LAD	2.40 (1.57, 3.67)	**<0.001**
PCI	0.22 (0.14, 0.34)	**<0.001**
OCT guidance	0.94 (0.64, 1.38)	0.752

CAD, coronary artery disease; LAD, left anterior descending artery; LVEF, left ventricular ejection fraction; MI, myocardial infarction; OCT, optical coherence tomography; PCI, percutaneous coronary intervention; µQFR, Murray-law based quantitative flow ratio.

Supplemental Table S3. Predictors of 3-year TVF

Variables	HR (95% CI)	p	Proportional hazard test p
Age, years	1.004 (0.97, 1.04)	0.809	0.42
Female	0.76 (0.31, 1.86)	0.547	0.22
Body mass index, kg/m^2	1.03 (0.95, 1.11)	0.535	0.44
Multivessel disease	0.54 (0.22, 1.33)	0.181	0.39
LVEF	0.998 (0.95, 1.04)	0.927	0.36
Diabetes mellitus	1.66 (0.81, 3.43)	0.167	0.86
Insulin dependent Diabetes mellitus	0.76 (0.10, 5.58)	0.787	0.38
Hypertension	1.62 (0.49, 5.35)	0.426	0.84
Dyslipidemia	0.85 (0.40, 1.81)	0.672	0.65
Smoking	0.79 (0.37, 1.68)	0.539	0.68
Chronic kidney disease	1.33 (0.57, 3.11)	0.505	0.52
Previous PCI	1.25 (0.61, 2.57)	0.535	0.37
Previous MI	0.45 (0.16, 1.28)	0.132	0.71
Family history of CAD	0.44 (0.17, 1.14)	0.091	0.76
Stable CAD	0.62 (0.28, 1.40)	0.249	0.38
Interrogated lesion in LAD	2.14 (0.87, 5.23)	0.096	0.16
PCI	0.58 (0.26, 1.31)	0.190	0.22
OCT guidance	0.24 (0.05, 1.13)	0.071	0.12
Final µQFR (per 0.10 increase) (baseline for deferred vessels and post-PCI for stented vessels)	0.55 (0.30, 0.98)	**0.044**	0.89

CAD, coronary artery disease; LAD, left anterior descending artery; LVEF, left ventricular ejection fraction; MI, myocardial infarction; OCT, optical coherence tomography; PCI, percutaneous coronary intervention; µQFR, Murray-law based quantitative flow ratio.

Supplemental Table S4. Sensitivity analysis of final µQFR in predicting 3-year TVF and TVR

	Adjusted HR (95% CI) *	P	Proportional hazard test p
**TVF**			
Model 1	4.50 (1.70, 11.90)	**0.002**	0.54
Model 2	2.96 (1.29, 6.75)	**0.010**	0.54
**TVR**			
Model 1	4.57 (1.71, 12.25)	**0.002**	0.77
Model 2	4.64 (1.69, 12.69)	**0.003**	0.57

*HR>1 favours final µQFR>0.89. The HRs represent the relative outcomes of vessels with final µQFR ≤0.89 compared with those with final µQFR >0.89. Analyses were performed by multivariable Cox regression in two models: 1) model 1 included patient ID as a random effect (random intercept); 2) model 2 included baseline covariates (interrogated lesion in LAD, age, female sex, diabetes mellitus and acute coronary syndrome) as fixed effects and patient ID as a random effect.

HR, hazard ratio; TVF, target vessel failure; TVR, target vessel revascularization; µQFR, Murray-law based quantitative flow ratio.

Supplemental Table S5. 3-year clinical outcomes in high and low final µQFR groups stratified by randomization arm

**FFR group (n = 217)**					
	**µQFR≤0.89 (n=102)**	**µQFR>0.89 (n=115)**	**HR (95% CI) ***	**p**	**Proportional hazard test p**
TVF	13 (12.8%)	4 (3.5%)	3.84 (1.25, 11.78)	**0.019**	0.46
Cardiac death	3 (2.9%)	1 (0.9%)	3.36 (0.35, 32.35)	0.293	0.25
TVMI	2 (2.0%)	3 (2.6%)	0.76 (0.13, 4.53)	0.760	0.49
TVR	11 (10.8%)	1 (0.9%)	13.08 (1.69, 101.36)	**0.014**	0.95
Cardiac death or TVMI	5 (4.9%)	4 (3.5%)	1.41 (0.38, 5.25)	0.609	0.77
**OCT group (n = 198)**					
	**µQFR≤0.89 (n=90)**	**µQFR>0.89 (n=108)**	**HR (95% CI) ***	**P**	**Proportional hazard test p**
TVF	9 (10.0%)	4 (3.7%)	2.74 (0.84, 8.89)	0.094	0.35
Cardiac death	0 (0.0%)	0 (0.0%)	-	-	-
TVMI	1 (1.1%)	1 (0.9%)	1.19 (0.07, 19.04)	0.902	0.16
TVR	8 (8.9%)	4 (3.7%)	2.42 (0.73, 8.05)	0.148	0.42
Cardiac death or TVMI	1 (1.1%)	1 (0.9%)	1.19 (0.07, 19.04)	0.902	0.16

*HR>1 favours final µQFR>0.89. Event rates were estimated from Kaplan-Meier analysis. FFR, fractional flow reserve; HR, hazard ratio; OCT, optical coherence tomography; PCI, percutaneous coronary intervention; TVF, target vessel failure; TVMI, target-vessel myocardial infarction; TVR, target vessel revascularization; µQFR, Murray-law based quantitative flow ratio.

Supplemental Table S6. 3-year clinical outcomes in high and low final µQFR groups stratified by vessel treatment

**Deferred vessels (n = 256)**					
	**µQFR≤0.89 (n = 153)**	**µQFR>0.89 (n = 103)**	**HR (95% CI) ***	**p**	**Proportional hazard test p**
TVF	19 (12.4%)	3 (2.9%)	4.47 (1.32, 15.10)	**0.016**	0.76
Cardiac death	3 (2.0%)	1 (1.0%)	2.03 (0.21, 19.52)	0.540	0.25
TVMI	3 (2.0%)	1 (1.0%)	2.03 (0.21, 19.52)	0.540	0.24
TVR	16 (10.5%)	1 (1.0%)	11.34 (1.50, 85.55)	**0.018**	0.97
Cardiac death or TVMI	6 (3.9%)	2 (1.9%)	2.04 (0.41, 10.09)	0.384	0.50
**Treated vessels (n = 159)**					
	**µQFR≤0.89 (n = 39)**	**µQFR>0.89 (n = 120)**	**HR (95% CI) ***	**p**	**Proportional hazard test p**
TVF	3 (7.7%)	5 (4.2%)	1.85 (0.44, 7.74)	0.400	0.57
Cardiac death	0 (0.0%)	0 (0.0%)	-	-	-
TVMI	0 (0.0%)	3 (2.5%)	-	-	-
TVR	3 (7.7%)	4 (3.3%)	2.32 (0.52, 10.39)	0.269	0.44
Cardiac death or TVMI	0 (0.0%)	3 (2.5%)	-	-	-

*HR>1 favours final µQFR>0.89. Event rates were estimated from Kaplan-Meier analysis. FFR, fractional flow reserve; HR, hazard ratio; OCT, optical coherence tomography; PCI, percutaneous coronary intervention; TVF, target vessel failure; TVMI, target-vessel myocardial infarction; TVR, target vessel revascularization.

Supplemental Table S7. Landmark analysis at 13-months for final µQFR ≤0.89 in predicting 3-year TVF and separate endpoints at a vessel-level

	**At 13-month**		**From 13-month to 3-year**		**P for interaction**
	**HR (95% CI) ***	**P**	**HR (95% CI) ***	**p**	
TVF	3.24 (1.03, 10.19)	**0.044**	3.34 (1.06, 10.49)	**0.039**	0.60
Cardiac death	-	-	1.16 (0.07, 18.57)	0.916	-
TVMI	0.39 (0.04, 3.71)	0.410	2.30 (0.21, 25.35)	0.497	0.04
TVR	5.31 (1.15, 24.59)	**0.033**	4.07 (1.12, 14.81)	**0.033**	0.20
Cardiac death or TVMI	1.16 (0.23, 5.73)	0.859	1.73 (0.29, 10.35)	0.549	0.09

*HR>1 favours final µQFR>0.89. Event rates were estimated from Kaplan-Meier analysis.

HR, hazard ratio; TVF, target vessel failure; TVMI, target-vessel myocardial infarction; TVR, target vessel revascularization.
